# A clustering-optimized segmentation algorithm and application on food quality detection

**DOI:** 10.1038/s41598-023-36309-8

**Published:** 2023-06-05

**Authors:** QingE Wu, Penglei Li, Zhiwu Chen, Tao Zong

**Affiliations:** School of Electrical and Information Engineering, Zhengzhou University of Light Industry, No. 5 Dongfeng Road, Jinshui District, Zhengzhou City, 450002 Henan Province China

**Keywords:** Computer science, Information technology

## Abstract

For solving the problem of quality detection in the production and processing of stuffed food, this paper suggests a small neighborhood clustering algorithm to segment the frozen dumpling image on the conveyor belt, which can effectively improve the qualified rate of food quality. This method builds feature vectors by obtaining the image's attribute parameters. The image is segmented by a distance function between categories using a small neighborhood clustering algorithm based on sample feature vectors to calculate the cluster centers. Moreover, this paper gives the selection of optimal segmentation points and sampling rate, calculates the optimal sampling rate, suggests a search method for optimal sampling rate, as well as a validity judgment function for segmentation. Optimized small neighborhood clustering (OSNC) algorithm uses the fast frozen dumpling image as a sample for continuous image target segmentation experiments. The experimental results show the accuracy of defect detection of OSNC algorithm is 95.9%. Compared with other existing segmentation algorithms, OSNC algorithm has stronger anti-interference ability, faster segmentation speed as well as more efficiently saves key information ability. It can effectively improve some disadvantages of other segmentation algorithms.

## Introduction

For effective segmentation so as to improve product qualification of defect targets, some hidden defect features, such as cracks, breakages, stains and so on need to be carried out detect early from images of food products containing fillings, which can greatly improve the recognition rate of dumpling defects, thus the quality of dumplings is approved. The image analysis of filled food products has become important with advances in artificial intelligence and increasing labor costs. The basis for image segmentation target recognition, matching and tracking were important for image understanding, image analysis, pattern recognition, computer vision and others^1^.

The result of image segmentation can segment a food processing scene image into target regions, thus providing the location of the target in the image. Algorithms based on grey-scale threshold segmentation^2^, edge segmentation^3^ and region segmentation^4^ are widely used in image segmentation. The threshold segmentation method is particularly suitable for images where the target and background occupy different grey level ranges and has been applied in many fields, where the selection of threshold values is a key technique in image threshold^5,6^. The edge information is the detailed information when the grey scale of the image changes^7^. In order to segment out the region of interest, there were other edge detection operators such as Sobel et al.^8^. The processing principle of such edge detection algorithms was used to record the grey jump, and when the grey jump matches the set threshold, the edge features are extracted using the difference operation method^9^. It has been shown that both segmentation methods are widely used. Yang^10^ proposed a supervised multiple threshold segmentation models to complete the detection of potato sprouting. In addition, scholars have also actively improved the edge detection operator. Lu^11^ introduced a threshold selection method based on the local maximum inter-class variance algorithm in the Canny edge detection algorithm in order to improve the efficiency of thermal image recognition. Liao^12^ used a supervised block-based region segmentation algorithm to segment tumor regions from breast ultrasound images, combined with a deep learning network, in order to predict whether a breast tumor is benign or malignant.

The cluster segmentation is one of the specific theoretical approaches to image, typically, the K-means clustering algorithm^13^ and the fuzzy C-mean clustering algorithm^14^. Trivedi^15^ used a K-means clustering segmentation algorithm to segment plant leaves into homogeneous segments which significantly improved the accuracy of plant leaf pest detection. Wu^16^ used the Canny algorithm to process text image edge detection and then used the k-means algorithm for clustering pixel recognition, which effectively improved the accuracy of text image recognition. Fuzzy C-Means (FCM) was the most useful image segmentation algorithm for realistic scenarios^17^. The FCM segmentation algorithm deals directly with the greyscale image by using fuzzy theory. The purpose of the clustering operation carried out classifying the dataset more accurately and reasonably, classifying all samples with similar features. Some samples with more different features could be classified in different categories so as to reach the most reasonable segmentation effect^18^. Gao^19^ proposed a robust fuzzy c-mean clustering method based on the adaptive elastic distance for image segmentation. Brikh^20^ combined fuzzy C-means and particle swarm optimization (PSO) algorithms to cluster large nonlinear data sets.

FCM clustering algorithm and K-means segmentation algorithm were well applied in Various image segmentation practices^21^. However, they also suffer from the following disadvantages, such as searching time of both types of algorithms and their derivatives is longer especially multi-threshold segmentation. The larger the image size is, the longer the segmentation time is^22^. The parameters need to be set, and the optimal number of partitions could not be obtained by existing methods^23^. In addition, because the defects of stuffed food are relatively small to obtain feature of defects difficultly. it is necessary to establish an algorithm suitable for this defect to realize dumpling image segmentation. This paper proposes an optimized small neighborhood clustering (OSNC) segmentation algorithm, which implements the segmentation of stuffed food, and verifies the effectiveness of the algorithm by using the open-source datasets.

**Figure 1:**
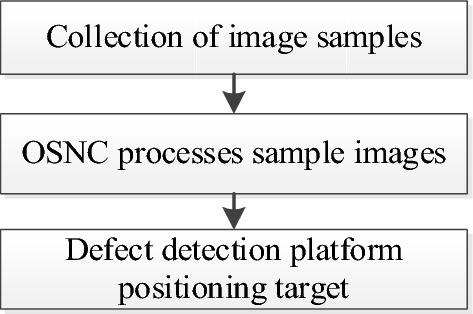
Flow chart of the frozen dumpling defect detection.

To verify the feasibility of the OSNC segmentation algorithm for fast and accurate segmentation of images of stuffed food in real production. Based on that, a Matlab defect detection platform was built to detect defects in the production process of frozen dumplings. The processes are as follows: (1) a grey-scale camera is set up to capture the image information of the frozen dumplings; (2) the OSNC algorithm pre-processes the samples; and (3) the defect detection platform locates the defective dumplings. The specific flow chart is as follow in Fig. 1.

## Small neighborhood clustering segmentation algorithm

Suppose $n$ is classes of sample data, each of which is a set of data $\alpha_{1} ,\alpha_{2} , \ldots ,\alpha_{n}$. Suppose that there are $m$ attribute parameters of the sample, For example, the image peak, single peak, grayscale, valley, color, edge, inflection point, tone, multimodal and other indicators^24^. The $m$-dimensional feature space is constructed according to the indicators of these sample points, and all the sample points correspond to the points in the $m$-dimensional feature space.

Small neighborhood segmentation algorithm is given as follows:

Step 1 For a given sample $x = \left\langle {c_{1} \left( x \right),c_{2} \left( x \right), \ldots ,c_{m} \left( x \right)} \right\rangle$, there are $m$ attributes for sample $x$.
Step 2 The $c_{q} \left( x \right)$, $q = 1,2, \ldots ,m$ is the $q{\text{ - th}}$ attribute about $x$.
Step 3 By calculating the distance between any two samples in the sample space, there are $K$ classes are obtained, that is closest to sample $x$, $y_{1} ,y_{1} , \ldots ,y_{K}$. $y_{i}$ represents one of the $K$.
Step 4 Regard class $x$ as a center, an appropriate class are found in a small neighborhood with radius $\varepsilon$ by clustering.
Step 5 For the sample $x$ that needs to be segmented, $d_{1} ,d_{2} , \cdots ,d_{K}$ between the $K$ nearest neighbors $\left( {x,y_{1} } \right)$, …, $\left( {x,y_{K} } \right)$ is defined by the distance.
Step 6 For $L$ samples $x$, that is marked as $x_{l}$, $l = 1,2, \ldots ,L$, and $n$ classes $\alpha_{i}$, $i = 1,2, \ldots ,n$, there are $N_{i}$ samples in each class, and one sample has $m$ attributes.
Step 7 Next, for $K$ neighbors of sample $x_{l}$, that is marked as $x_{l + 1} , \ldots ,x_{l + K}$, the center of single attributes is shown under the formula.

For sample $x$ the same attribute of $K$ that is marked as $x_{l + 1} , \ldots ,x_{l + K}$ of is defined as follows:

1 $$V_{l}^{q} = \frac{1}{K + 1}\sum\limits_{j = l}^{K + l} {c_{q} \left( {x_{j} } \right)} , \, q = 1,2, \ldots ,m, \, l = 1,2, \ldots ,L$$

The small neighborhood clustering algorithm further can be divided into two stages: training stage and segmentation stage.

The training procedures are as follows:

1. Configure the original iterative value of the algorithm to the same attribute, and set the sample number $s$ to $s = 0$.
2. Then the circle center attribute is set to $V_{l}^{q}$. Compute the nearest neighbors of $V_{l}^{q}$ in a small neighborhood of radius $\varepsilon$. When you get a proper nearest neighbor $V$, update the value of $s$: $s = s + 1$.
3. Search P2 in turn until the nearest neighbor $V$ does not exist, $s$ neighbors of $V_{l}^{q}$ can be obtained. Then the number of samples of the same attribute is $s$. Then, the weight of each attribute of the sample can be defined as $\zeta_{q} = {s \mathord{\left/ {\vphantom {s L}} \right. \kern-0pt} L}$,$q = 1,2, \ldots ,m$. Define $\zeta_{p} = \mathop {\max }\limits_{{q \in \left\{ {1,2, \cdots ,m} \right\}}} \left\{ {\zeta_{q} } \right\}$, Then $\zeta_{p}$ is the known class. Combined with formula (1), the average value of the same attribute for different samples of each class is defined as follows:
  2 $$V_{{\alpha_{i} }}^{q} = \frac{1}{{N_{i} }}\sum\limits_{l = 1}^{{N_{i} }} {c_{q} \left( {x_{l} } \right)} , \, q = 1,2, \ldots ,m, \, i = 1,2, \ldots ,n$$

**Figure 2:**
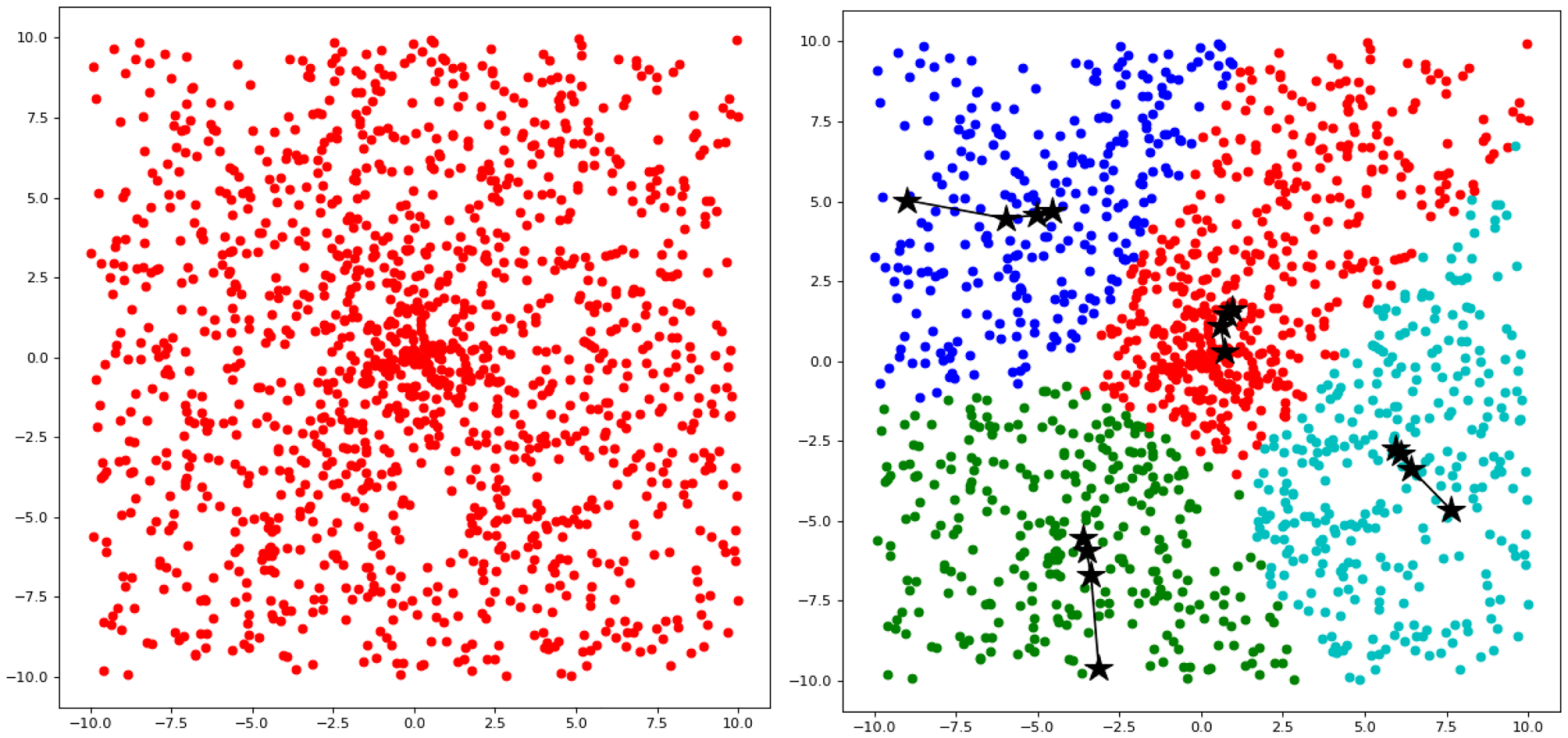
Small neighborhood clustering process and trajectory of clustering centers.

The training process is shown in Fig. 2.

The segmentation procedures are as follows:

1. First set $$c_{h0} = 0$$, $$b_{hq} = 0$$.
2. Set $N_{i}$ to a certain value $z$.
3. Set the iteration initial value $v = 0$.
4. Set the center to $V_{{\alpha_{i} }}^{q}$. Search the nearest neighbor $V^{\prime}$ of $V_{{\alpha_{i} }}^{q}$ in a small neighborhood with radius $\varepsilon$. Whenever new $V^{\prime}$ is searched, update the value of $v$: $v = v + 1$.
5. According to the above P4 search small neighborhood algorithm, simulation iteration, until the nearest neighbor $V^{\prime}$ does not exist.
6. Given $$z = z + 1$$.
  At the same time, the number of obtained $V_{{\alpha_{i} }}^{q}$ is stored as $v$ and assigned to $c_{h0} = \max \left\{ {c_{h0} ,v} \right\}$. Then, the weight of each attribute corresponding to the sample contained in each class can be defined as ${v \mathord{\left/ {\vphantom {v {N_{i} }}} \right. \kern-0pt} {N_{i} }}$ and assigned to $b_{hq} = \max \left\{ {b_{hq} ,{v \mathord{\left/ {\vphantom {v {N_{i} }}} \right. \kern-0pt} {N_{i} }}} \right\}$,$q = 1,2, \ldots ,m$, $h = 1,2, \ldots ,N_{i}$.
7. If $v = c_{h0}$, the iteration is terminated. Otherwise, P3 ~ P6 are continued until $v = c_{h0}$, and the segmentation ends. At this time, the number of total classes of the dataset to be segmented is $z$, and assigned to $n = z$. In this way, the dataset is first divided into $n$ classes. At the same time, assigned to $c_{0} = c_{h0}$ and $b_{q} = b_{hq}$.

Suppose that the data to be segmented has been determined $n$ classes, and each class has $m$ mean. The metric function $d_{i}$ of the distance function between the sample element $x$ to be segmented and a certain type of element in the training sample is:

3 $$d_{i} \left( {x,\alpha_{i} } \right) = \sum\limits_{q = 1}^{m} {b_{q} } \frac{{\left| {c_{q} \left( x \right) - V_{{\alpha_{i} }}^{q} } \right|}}{{O_{iq}^{u} - O_{iq}^{s} }}$$

where, $$O_{iq}^{s} = \mathop {\min }\limits_{{h \in \left\{ {1,2, \cdots ,N_{i} } \right\}}} \left\{ {c_{q} \left( x \right),c_{q} \left( {x_{h} } \right)} \right\}$$, $$O_{iq}^{u} = \mathop {\max }\limits_{{r \in \left\{ {1,2, \cdots ,N_{i} } \right\}}} \left\{ {c_{q} \left( x \right),c_{q} \left( {x_{h} } \right)} \right\}$$, $$i = 1,2, \ldots ,n$$.

Then the minimum values of these distances are obtained as shown in Formula (4):

4 $$d_{i * } \left( {x,\alpha_{i*} } \right) = \mathop {\min }\limits_{{i \in \left\{ {1,2, \cdots ,n} \right\}}} \left\{ {d_{i} \left( {x,\alpha_{i} } \right)} \right\}$$

Then the formula (4) can determine which class $\alpha_{i*}$ the sample $x$ to be segmented belongs to. For the sample $x$ to be segmented, the distances between $x$ and $n$ classes are respectively defined as $d_{1} ,d_{2} , \ldots ,d_{n}$. The calculation of decision weight can also be calculated by $\lambda_{i}$ as shown in Formula (5):

5 $$\lambda_{i} = \frac{1}{n - 1}\left( {1 - \frac{{d_{i} }}{{\sum\limits_{i = 1}^{n} {d_{i} } }}} \right)$$

**Figure 3:**
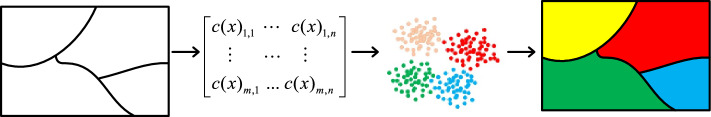
Image feature clustering segmentation based on small neighborhood clustering.

Satisfying $\sum\nolimits_{i = 1}^{n} {\lambda_{i} = 1}$. Define $\lambda_{i * } = \mathop {\max }\limits_{{i \in \left\{ {1,2, \cdots ,n} \right\}}} \left\{ {\lambda_{i} } \right\}$. From the obtained $i *$, which can also determine which class $\alpha_{i*}$ the sample $x$ to be segmented belongs to. The segmentation algorithm is shown in Fig. 3.

## Optimistic method

### Selection of optimal segmentation points

The pixel value of the grayscale image is used as the input of the algorithm to verify the effective segmentation algorithm. If the shape of the image is $M \times N$, the corresponding image grayscale value matrix set is $L = \left\{ {{\mathbf{L}}_{{{\mathbf{ij}}}} ,i = 1,2, \ldots ,M,j = 1,2, \ldots ,N} \right\}$. Define the set of its segmentation centers as $O = \{ o_{k} ,k = 1,2, \ldots ,n\}$. $U = \{ \mu_{k} ({\mathbf{L}}_{{{\mathbf{ij}}}} )\}$ is the membership set of pixels $(i,j)$ in the defined class $k$, and $D = \{ d_{ijk} ,k = 1,2, \ldots ,n,i = 1,2, \ldots ,M,j = 1,2, \ldots ,N\}$ are the set of distances between cluster centers. The objective function formula of segmentation center is:

6 $$B_{f} = \sum\limits_{k = 1}^{n} {\sum\limits_{i = 1}^{M} {\sum\limits_{j = 1}^{N} {[\mu_{k} ({\mathbf{L}}_{{{\mathbf{ij}}}} )]^{r} (d_{ijk} )^{2} } } } = \sum {U(i,j,k)^{r} D(i,j,k)^{2} }$$

where $r$ is the fuzzy weight index. There is:

7 $$\sum\limits_{k = 1}^{n} {\mu_{k} ({\mathbf{L}}_{{{\mathbf{ij}}}} )} = 1$$

The calculation results of the segmentation center $o_{k}$ and the final value $\mu_{k} ({\mathbf{L}}_{ij} )$ of the membership matrix are shown in Formulas (8) and (9):

8 $$o_{k} = \frac{{\sum {U(i,j,k)_{{}}^{r} {\mathbf{L}}(i,j)} }}{{\sum {U(i,j,k)_{{}}^{m} } }},k = 1,2, \ldots ,n$$

9 $$\mu_{k} ({\mathbf{L}}_{{{\mathbf{ij}}}} ) = \frac{{D(i,j,k)^{{ - \frac{2}{r - 1}}} }}{{\sum\limits_{k = 1}^{n} {D(i,j,k)^{{ - \frac{2}{r - 1}}} } }},k = 1,2, \ldots ,n$$

Segmentation center can be calculated quickly by initial membership matrix and formula (8). Then calculate the new value of $\mu_{k} ({\mathbf{L}}_{{{\mathbf{ij}}}} )(\forall k,i,j)$ by $o_{k}$ and formula (9). After many calculations, until $\mu_{k} ({\mathbf{L}}_{{{\mathbf{ij}}}} )(\forall k,i,j)$ is stable. Define $O$ as the final set of segmentation centers and use the following formula to calculate the image segmentation threshold:

10 $$J_{c} = \beta o_{c} + \tilde{\beta }o_{c + 1} ,c = 1,2, \ldots ,G$$

where, $G$ is the number of thresholds, $\beta$ and $\tilde{\beta }$ are the weight coefficients. satisfy the formula (11).

11 $$\beta + \tilde{\beta } = 1$$

Usually select $$\beta = \tilde{\beta } = 0.5$$.

**Figure 4:**
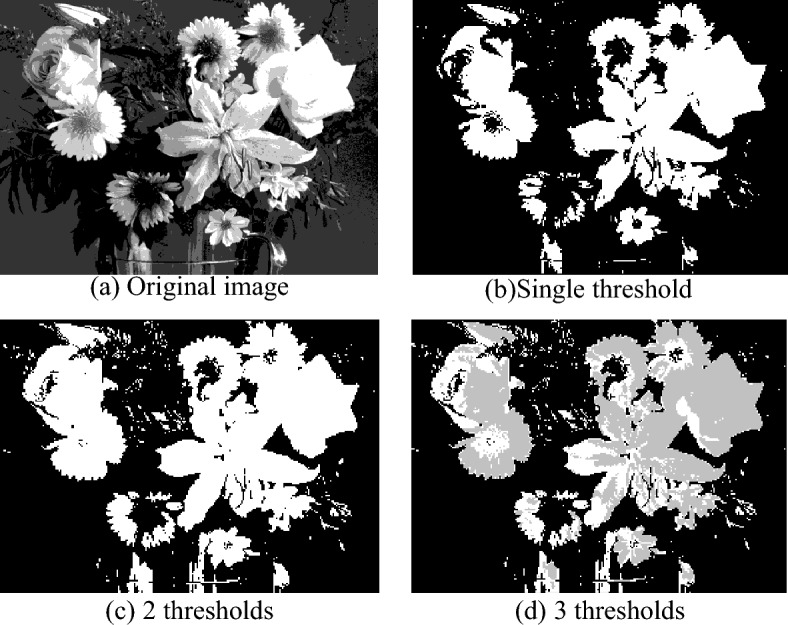
Results of different threshold segmentation.

This paper takes pictures in VOC database as segmentation samples. The above segmentation algorithm is used to segment the testing image with different thresholds, and the results are shown in Fig. 4.

### Selection of optimal segmentation sampling rate

Usually, the fixed interval algorithm for image information acquisition does not have much impact on the image processing results, and can save equipment memory. Therefore, most image processing algorithms will resample the image. The resampling algorithm can be described as formula (12):

12 $$\left[ {\begin{array}{*{20}c} {x_{1} } \\ {y_{1} } \\ 1 \\ \end{array} } \right] = \left[ {\begin{array}{*{20}c} \eta & 0 & 0 \\ 0 & \eta & 0 \\ 0 & 0 & 1 \\ \end{array} } \right]\left[ {\begin{array}{*{20}c} {x_{0} } \\ {y_{0} } \\ 1 \\ \end{array} } \right]$$

The value range of resampling rate is $0 < \eta < 1$, the coordinate of initial image is $(x_{0} ,y_{0} )$, and it is $(x_{1} ,y_{1} )$ after formula transformation. The new data generated is related to the value of $\eta$. When the value of $\eta$ is small, the information acquisition effect is good, but the image distortion is obvious, and important information is lost. Therefore, selecting appropriate proportion is the key to effective segmentation. Selecting the appropriate sampling rate can make the information loss acceptable, which is a feasible algorithm. The information calculation referred to the segmentation method based on histogram entropy.

#### Calculation of optimal sampling rate

**Figure 5:**
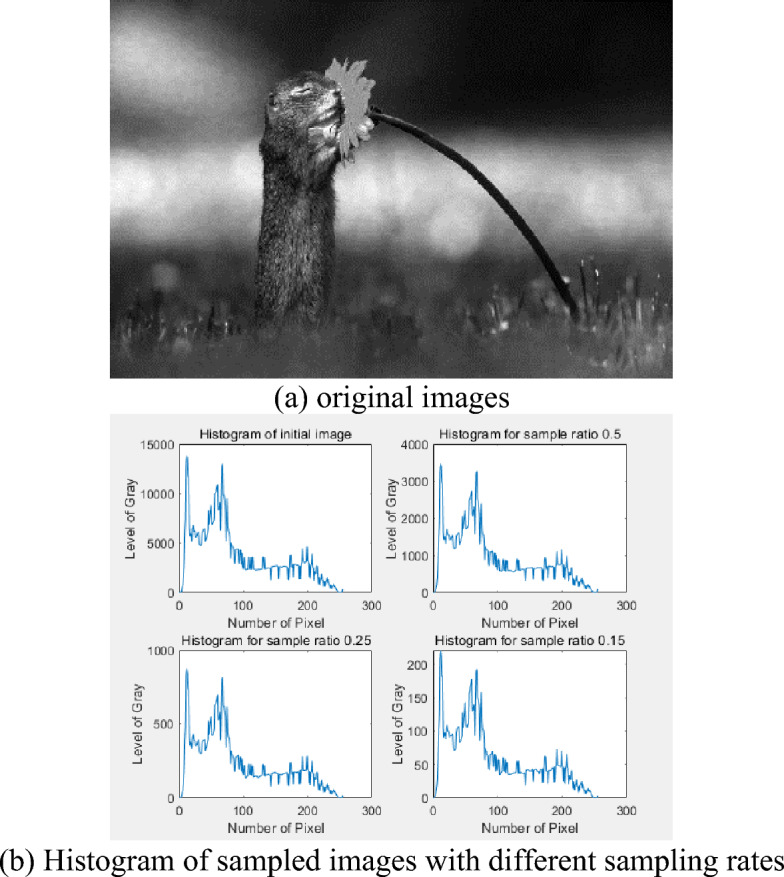
Histogram of the image.

The algorithm proposed in this paper uses entropy loss information as the standard to evaluate the distortion degree of the image. On this basis, in order to achieve good segmentation effect, the relative entropy loss degree is used as the selection basis of sampling rate in sampling. When the sample image has enough segmentation information, the sample image information is used to calculate the segmentation threshold. The obtained sample image is similar to the histogram shape of the original image, that is, the information of the sample image is basically the same as that of the original image. Figure 5 shows the sample image and its histogram at different sampling rates.

According to Fig. 5, the histogram shape of the sample image^25^ is basically the same under different resampling rates, indicating that the resampled image retains most of the information of the original image; But histograms differ from each other. When the sampling rate decreases, the image information is lost, and the curves in each histogram change obviously, which indicates that the accuracy of segmentation can be guaranteed by obtaining appropriate sampling rate.

The definition of Shannon entropy is shown in formula (13):

13 $$S = - \sum\limits_{i = 1}^{n} {P(\omega_{i} |x)} \log P(\omega_{i} |x)$$

where, $n$ is the class number, $x$ is the image feature, $\omega_{i}$ represents the $i$ class. For images of size $M \times N$, define information entropy as shown in formula (14):

14 $$S = - \sum\limits_{k = 0}^{G - 1} {P_{k} \log P_{k} }$$

Define $$P_{k}$$ as:

15 $$P_{k} = \frac{1}{MN}\sum\limits_{i = 0}^{M - 1} {\sum\limits_{j = 0}^{N - 1} {\rho_{ij} (k)} }$$

16 $$\rho_{ij} (k) = \left\{ {\begin{array}{*{20}l} 1 \hfill & {R(i,j) = k} \hfill \\ 0 \hfill & {{\text{else}}} \hfill \\ \end{array} } \right.,k = 1, \ldots ,C - 1$$

where, $C$ is the sum of grayscale levels, and $R(i,j)$ is the grayscale value. $P_{k}$ satisfies the following:

17 $$\sum\limits_{k = 0}^{C - 1} {P_{k} } = 1$$

Relative entropy loss can measure the degree of information loss. Suppose that the entropy of the sample image is $S_{1}$, the entropy of the sample image is $S_{\eta }$ when the sampling rate is $\eta$, the relative entropy loss is as follows:

18 $$\delta_{\eta } = \left| {\frac{{S_{1} - S_{\eta } }}{{S_{1} }}} \right|$$

**Figure 6:**
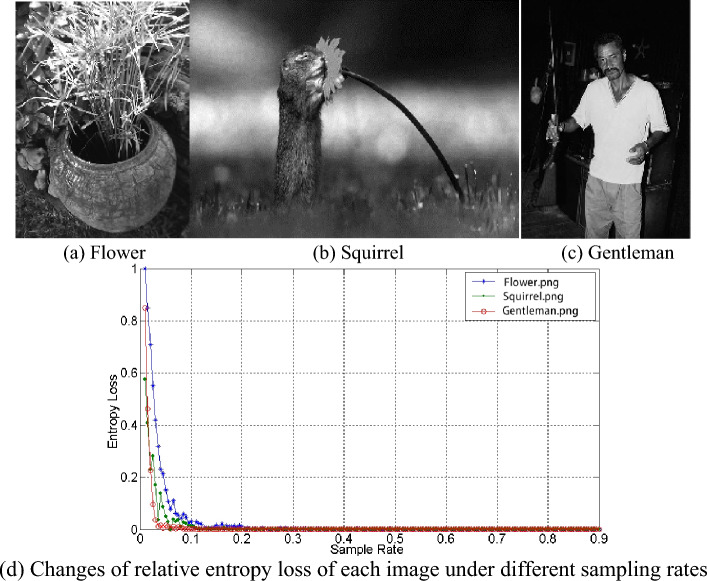
The relationship between different image sampling rates and relative entropy loss.

It can be seen from the above analysis that the relative entropy loss can be used as the basis for the selection of sampling rate. In order to explore the relationship between them, this paper analyzes the change trend of relative entropy loss in the range of sampling rate $\eta \in [0.01,0.9]$, and the trend curve is shown in Fig. 6.

Figure 6a–c are three different original images. Figure 6d shows that $\delta_{\eta }$ increases when $\eta$ decreases. This trend shows that when $\eta$ decreases, the sample image distortion increases, but the distortion is small. Most of the original information remains within a certain sampling rate. When the sampling rate is very small, $\delta_{\eta }$ will greatly increase. Within the allowable range of relative entropy loss, the image has less data at the minimum sampling rate and the thresholds calculated are also reliable. In this range, the minimum sampling rate can be calculated by searching the optimal sampling rate algorithm.

#### Search for optimal sampling rate

The minimum sampling rate can be calculated by dichotomy. Although this algorithm is proved to be effective, it needs more iterations. In order to improve the search efficiency, variable step search can be used to find the minimum sampling rate. Suppose the relative entropy loss range is $[\delta_{\min } ,\delta_{\max } ]$, the optimal sampling rate $\eta_{o}$ is:

19 $$\eta_{o} = \min \{ \eta |\delta_{\eta } \in [\delta_{\min } ,\delta_{\max } ]\}$$

In fact, $\eta_{o}$ cannot be calculated accurately. It is unnecessary to continuously search $\eta_{o}$ for the accuracy of this paper. Therefore, this paper selects the first sampling rate $\eta_{f}$ instead of $\eta_{o}$ to meet the constraint of relative entropy loss. Suppose that the current iterative search step is $t$, the variable step search algorithm is as follows:

20 $$\left\{ \begin{gathered} t = \eta k \hfill \\ \eta * = \eta - t \hfill \\ \end{gathered} \right.,\;\;\delta_{\eta } < \delta_{\min }$$

21 $$\left\{ \begin{gathered} t = \eta (1 - k/2) \hfill \\ \eta * = \eta + t \hfill \\ \end{gathered} \right.,\;\;\;\delta_{\eta } > \delta_{\max }$$

For a single target image, the number of sample image datasets with sampling rate $\eta_{f}$ is limited. Therefore, the histogram created cannot contain data for each class, which affects the single peak judgment. To calculate the optimal number of thresholds, use the size $M \times N$ of image $S_{0}$ to ensure that the sampling rate is within the optimal range. Therefore, the optimal sampling rate $\eta_{o}$ can be defined as:

22 $$\eta_{o} = \left\{ {\begin{array}{*{20}c} {\frac{{S_{0} }}{\min (M,N)},} & {\eta \le \frac{{S_{0} }}{\min (M,N)}} \\ {\eta_{f} ,} & {\eta_{f} > \frac{{S_{0} }}{\min (M,N)}} \\ \end{array} } \right.$$

Set the number of optimization steps $H$, the number of sample classes $(N + 1)$, the number of class separation distance $k$, so the complexity of the algorithm $\theta_{x}$ is as follows:

23 $$\theta_{x} = H \times (N + 1) \times k$$

### Judgment function of validity in segmentation

In this section, in order to find out the optimal segmentation number of images, an improved correlation function between fuzzy sets is constructed. This function is used to judge the effectiveness of image segmentation^26^. In fuzzy partition, fuzzy membership describes the correlation of classification data sets.

Suppose that the shape of the image is $M \times N$, the corresponding set $L = \left\{ {L_{ij} ,i = 1,2, \ldots ,M,j = 1,2, \ldots ,N} \right\}$ of image grayscale value matrices containing $\alpha$ classes.

Then the fuzzy deviation degree for class $c$ is:

24 $$\delta_{c}^{2} = \sum\limits_{i = 1}^{M} {\sum\limits_{j = 1}^{N} {\mu_{c}^{2} (L_{ij} )} } \left\| {L_{ij} - \nu_{c} } \right\|^{2}$$

Define the fuzzy relation matrix set $R_{kl}$ as:

25 $$R_{kl} = \sum\limits_{i = 1}^{M} {\sum\limits_{j = 1}^{N} {\mu_{k} (L_{ij} )\mu_{l} (L_{ij} )} } \left\| {L_{ij} - \nu_{k} } \right\|\left\| {L_{ij} - \nu_{l} } \right\|$$

The fuzzy membership of classes $k$ and $l$ is defined as:

26 $$\varphi_{kl} = \frac{{R_{kl} }}{{\delta_{k}^{{}} \delta_{l}^{{}} }}$$

Then fuzzy membership function can be defined as validity judgment function. If the following equation is satisfied:

27 $$F(U*,\alpha *) = \min \left\{ {\max \varphi_{kl} (U,\alpha )} \right\},1 \le k \le \alpha ,1 \le l \le \alpha ,l \ne k$$

Then $\alpha *$ is the optimal segmentation number of the sample image.

**Figure 7:**
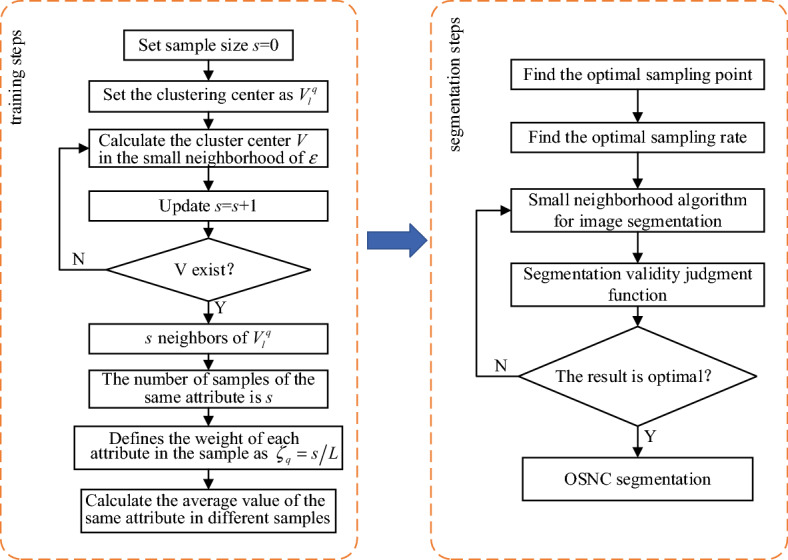
Flow chart of OSNC algorithm.

Through the above analysis, the flow chart of the OSNC algorithm constructed in this paper is shown in Fig. 7.

## Evaluation index

Establish a unified comparative value: Supposing that the number of testing samples in a single experiment is $N$ and the number of correct detections is $n_{i} (i = 1,2, \ldots ,N)$, the single recognition accuracy rate $M_{s}$ is defined as follows according to the experimental situation:

28 $$M_{s} = \frac{{n_{i} }}{N},(i = 1,2, \ldots ,N)$$

Suppose the number of experiments is $P$, the average recognition accuracy $M$ is:

29 $$M = \frac{{\sum {M_{s} } }}{P}$$

## Experiment and result analysis

The experiment environment is Windows 10 operating system, and all the simulation experiments are run using a CPU of Intel(R) Core(TM) i7-9700, a 4-core processor at 3.0 GHz, 32.0 GB RAM.

In order to verify the effectiveness of the segmentation validity judgment function. Taking Fig. 6a as the segmentation sample, the value of the segmentation validity judgment function is calculated. The experimental comparison results are shown in Table 1.

**Table 1:** Comparison results of iteration times and searching time between this paper and the other algorithm.

Number of segmentations		2	3	4	5	6	7	8	9
K-means	Iterative times	13	18	16	17	15	20	15	22
	Searching time (ms)	19	39	42	64	77	143	113	180
FCM	Iterative times	12	20	15	18	14	16	17	29
	Searching time (ms)	18	27	35	80	62	71	94	96
PSO-FCM	Iterative times	13	19	18	19	18	17	20	24
	Searching time (ms)	20	27	34	70	60	58	81	97
OSNC	Iterative times	10	11	13	12	13	11	10	9
	Searching time (ms)	18	20	31	66	65	70	100	92

**Figure 8:**
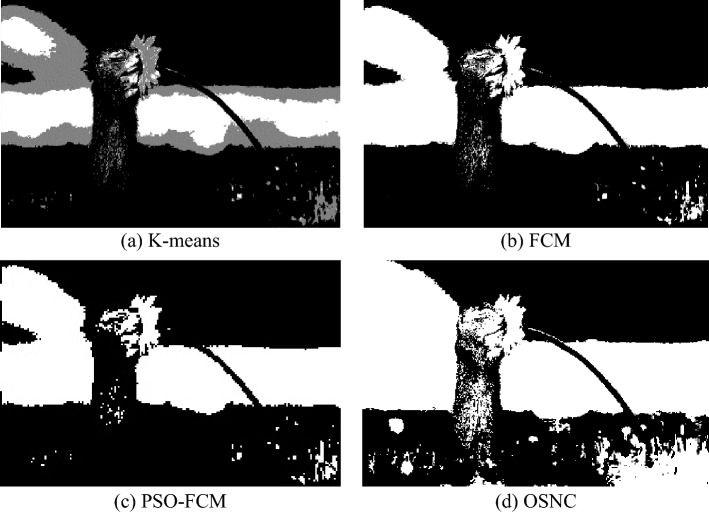
Comparison of segmentation results between proposed algorithm and other segmentation algorithm.

As shown in Table 1, the OSNC method has fewer iterations and shorter searching time than the other methods (K-means^15^, FCM^21^, PSO-FCM^20^). Experiments show that this method reduces the search time of the optimal segmentation number to a certain extent. The results of the four image segmentation methods are shown in Fig. 8.

This paper uses the sample images in the VOC database to verify the feasibility of the algorithm through continuous image segmentation experiments. Next, for the image samples collected in the industrial production site of frozen dumplings, the effectiveness of the OSNC algorithm is verified by combining the Matlab image processing platform.

## Comparison of OSNC algorithm and existing algorithms

### Data source

**Figure 9:**
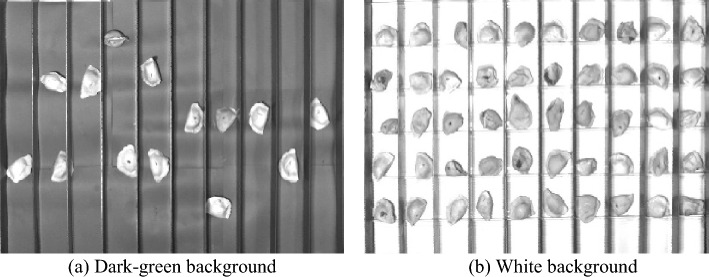
Sample images under different background.

In this paper, the effectiveness of the algorithm is verified by the field images data of the factory frozen dumpling production line. Sample images are sampled by grayscale camera. To ensure images quality, the resolution ratio of the camera reaches at least 2 million pixels. The camera is erected directly above the conveyor belt, and the receptive field cannot exceed the maximum edge of the conveyor belt. The camera samples every 0.15 s. Image samples include positive samples (qualified dumplings) and negative samples (defective dumplings), and normalized to the same size. Sample images captured under different background colors (dark-green and white) are shown in Fig. 9.

### Experiment

The hardware environment of the experiment is Windows 10 operating system, and all the simulation experiments are run using a CPU of Intel(R) Core(TM) i7-9700, a 4-core processor at 3.0 GHz, 32.0 GB RAM. The software of this experiment is a YOLOv3 defect detection platform based on Matlab, and the OSNC image segmentation algorithm is added after the input and before the backbone network.

In this experiment, 4000 images of frozen dumplings were used as sample databases, including 2000 images of dark-green background and 2000 images of white background. The database is divided into training samples and testing samples according to the ratio of 1:1.

**P1**: In the training sample, all experiments used the restriction $\delta \in [0.01,0.02]$, and the single-peak determination threshold $\xi_{h} = 0.015$.

**Figure 10:**
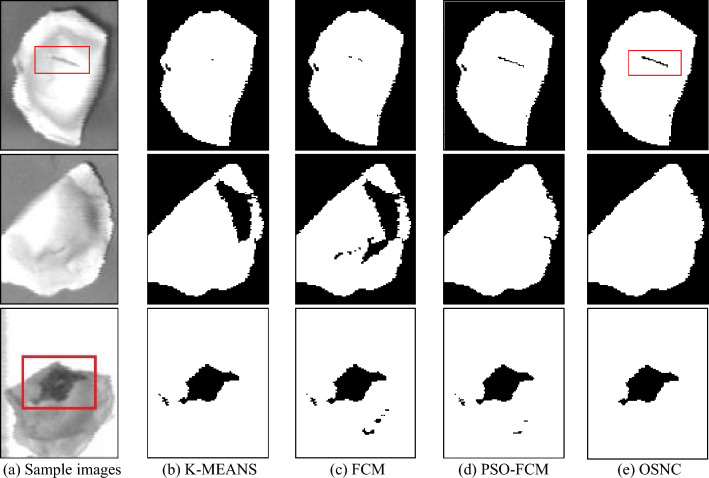
Segmentation results for simple image samples.

**Figure 11:**
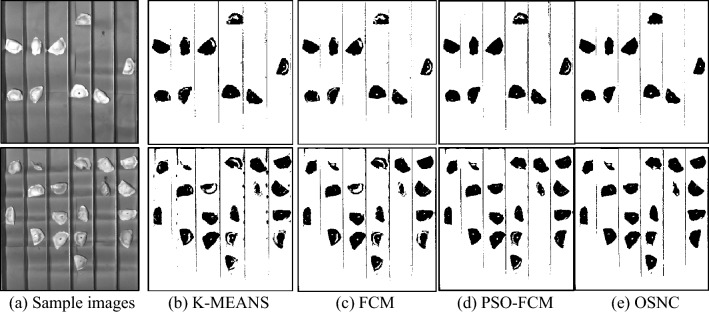
Segmentation results for complex image samples.

**P2**: Simple and complex sample images were segmented using the K-MEANS^15^, FCM^21^ and PSO-FCM^20^ segmentation algorithm and the OSNC segmentation algorithm proposed in this paper. The results are shown in Figs. 10 and 11.

Figure 10 contains samples of dumplings in different backgrounds; the sample images are of cracked surface dumplings, normal dumplings, and defective dumplings. In each row, the K-MEANS segmentation algorithm fails to segment the cracked defects, or is considered to be insensitive to changes in the grey value at the cracked defects, and uses the folds of the dumpling skin as key information for segmentation. the FCM segmentation algorithm is too sensitive to changes in the grey value of the overall image, and the segmentation contains both key and noisy information; PSO-FCM can effectively remove the background interference, but it will retain most of the defect information and redundant information at the same time. The PSO-FCM segmentation effect is better than the original FCM segmentation algorithm; the OSNC algorithm proposed in this paper is effective in segmenting the cracked dumplings for The OSNC algorithm proposed in this paper can effectively segment the key information of the dumpling cracks and is almost unaffected by noise, which provides a good preparation for subsequent defect detection. In Fig. 11, all four segmentation algorithms can effectively segment the background, defects and dumpling wrapper. However, in terms of segmentation effectiveness, the OSNC algorithm in this paper has significant background noise reduction, can retain the key dumpling defect features, and is highly resistant to interference.

**P3:** The training sample images processed by K-means, FCM, PSO-FCM and OSNC segmentation algorithms are respectively imported into Matlab image processing platform. After the model training is stable, the corresponding four models are recorded as: K-means, FCM, PSO-FCM and OSNC. The platform uses fast convolution network combined with edge detection algorithm for feature extraction. The dumplings that do not meet the production requirements such as surface damage, crack and stain can be identified and framed, and the label is defined as "Bad". For qualified dumplings can also be identified and framed, define the label as "Good".

**Figure 12:**
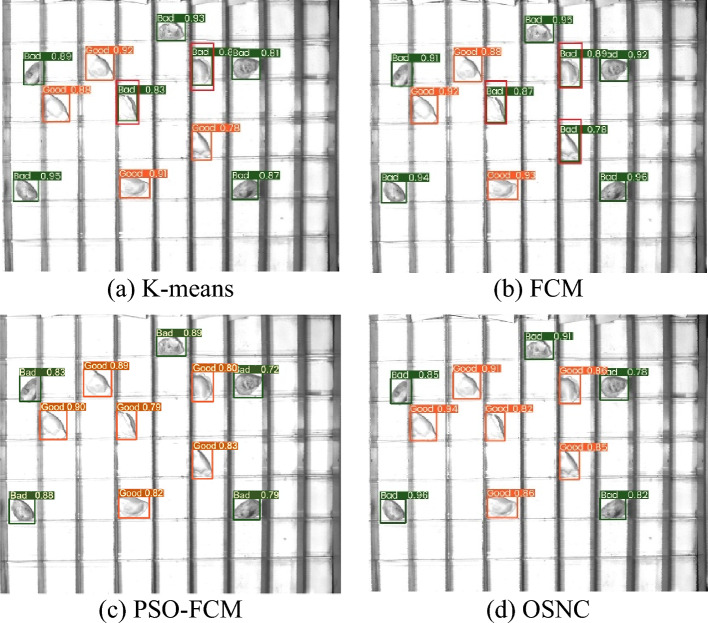
Comparison of detection effects of four models.

**P4:** Use testing sample images to test the defect detection effect of frozen dumplings. For the same test sample images, the visual detection results of the four models are shown in Fig. 12. According to Fig. 12, using the model of K-means, FCM and PSO-FCM segmentation algorithm, there are some misjudgments in dumpling defect detection, as shown in the red box in the Fig. 12. In contrast, the model using OSNC algorithm can accurately identify qualified and unqualified dumplings, and has stronger anti-interference ability and higher confidence level.

**P5:** Evaluate four defect detection methods. Four defect detection models obtained by P3 were used to detect all test sample images. 500 experiments were conducted respectively. The experiment records the detection time and the results of each model (the number of correct recognition and error recognition).

### Result analysis

**Figure 13:**
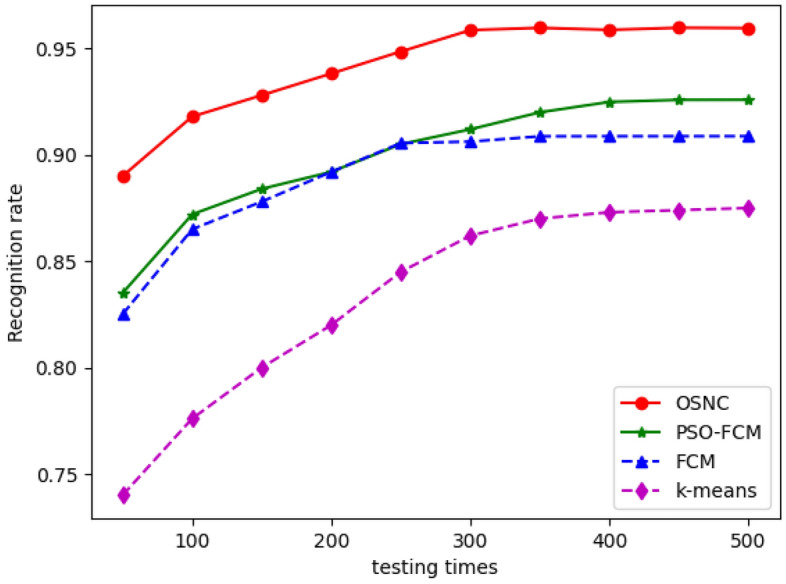
Comparison of recognition accuracy rate of models.

In the experiment, the recognition accuracy rate is calculated every 50 times, and the comparison results are shown in Fig. 13. The total number of samples, the number of accurately detected samples and the detection accuracy rate can be obtained from the Figure at any time. As shown in the Figure, with the increase of sample size, the accuracy of dumpling defect detection increases. After reaching a certain sample size, the curve tends to be stable. After 500 experiments, the accuracy rates of defect detection of frozen dumplings using the models of OSNC, PSO-FCM, FCM and K-means algorithms were 95.9%, 92.5%, 90.2% and 87.5%, respectively. The experimental results show that the OSNC algorithm can not only improve the accuracy rate of model defect detection, but also shorten the detection time.

Comprehensive evaluation of the performance of four segmentation algorithms: (1) The segmentation time of the algorithm for segmentation samples. For the training samples of this experiment, it is the average time for the algorithm to segment samples 10 times. (2) Anti-interference capability of the algorithm. It is an approximate estimation based on the segmentation effect and algorithm complexity. (3) Defect detection time and recognition accuracy. For the above 500 defect detection experiments. The model completed the average time and average recognition accuracy rate of dumpling defect detection. The comprehensive comparison results are shown in Table 2.

**Table 2:** Comprehensive comparison of different segmentation algorithms.

Algorithms	Segmentation time (s)	Anti-interference ability	Defect detection time (s)	Detection accuracy rate (%)
K-means	25.46	weak	0.121	87.5
FCM	18.62	inferior	0.092	90.2
PSO-FCM	20.17	inferior	0.012	92.5
Proposed method	12.98	strong	0.053	95.9

From the comparison of experimental results, the OSNC algorithm can quickly and accurately segment the frozen dumpling images. The image detection model using OSNC algorithm not only has fast processing speed, but also has higher recognition accuracy for frozen dumpling defects, which is more than 5% higher than that using other segmentation algorithm. The effectiveness of the algorithm established in this paper is proved.

In addition, the OSNC algorithm has strong anti-interference ability and better adaptability to different environments. In order to further improve the accuracy rate of defect detection, in actual processing and production, two cameras can be used to sample the image information of the dumplings on the conveyor belt. It is convenient for subsequent executing agencies to eliminate unqualified dumplings.

## Discussion

In this paper, an OSNC segmentation algorithm is established to cluster the feature vectors of stuffed food images. The image is segmented by using the distance function between categories. In order to optimize the OSNC segmentation algorithm, this paper calculates the best segmentation point by constructing the objective function of the clustering segmentation center; the variable step search algorithm is introduced to optimize the time of calculating the minimum sampling rate and improve the segmentation speed. At the same time, the relative entropy loss is used as the basis for judging the image sampling distortion. In addition, the fuzzy correlation is also considered, and the validity judgment function of segmentation is obtained, and the optimal segmentation number can be calculated. This paper used the images in the VOC database to verify the feasibility of the algorithm, and used the frozen dumpling image to verify the effectiveness of the algorithm. According to the comparative experimental results, the OSNC algorithm has faster segmentation speed and stronger anti-interference ability. The defect detection accuracy rate of the image processing model using this algorithm is more than 95%, which is about 5% higher than that of the other algorithm, and the defect detection speed is faster. The application of this method can meet the factory 's detection and elimination of defective dumplings and improve the qualified rate of dumpling production.

In order to enhance the rapidity and robustness, the small neighborhood algorithm will be improved through the aspects of objective function, membership function and distance function, in the future research.

## Data Availability

The data that support the findings of this study are available on request from the corresponding author. The data are not publicly available due to privacy or ethical restrictions.

## Abbreviations

FCM: Fuzzy C-means clustering algorithm
K-Means: K-means clustering algorithm
PSO-FCM: Particle swarm optimization-fuzzy C-means clustering algorithm
OSNC: Optimized small neighborhood clustering algorithm
VOC: Visual object class dataset
